# First record of the genus *Medaura* Stål (Phasmatodea, Phasmatidae, Clitumninae) from China, with description of a new species

**DOI:** 10.3897/BDJ.10.e96341

**Published:** 2022-12-09

**Authors:** YuHan Qian, ChongXin Xie, Cui Li

**Affiliations:** 1 Key Laboratory for Forest Resources Conservation and Utilization in the Southwest Mountains of China, Ministry of Education, Southwest Forestry University, Kunming, Yunnan 650224, China Key Laboratory for Forest Resources Conservation and Utilization in the Southwest Mountains of China, Ministry of Education, Southwest Forestry University Kunming, Yunnan 650224 China; 2 Faculty of Biodiversity Conservation, Southwest Forestry University, Kunming, Yunnan 650224, China Faculty of Biodiversity Conservation, Southwest Forestry University Kunming, Yunnan 650224 China

**Keywords:** stick insect, Medaurini, new record, taxonomy, Yunnan Province

## Abstract

**Background:**

The stick insect tribe, Medaurini in subfamily Clitumniae, contains 11 genera and 75 known species, with species diversity of this tribe being rich in southeast Asia and China, as is reflected in the Phasmida Species File Online [PSF]. The genus *Medaura* includes four named species and they are distributed over Bangladesh, Indonesia and India. The discovery of this new species in Xishuangbanna Dai Autonomous Prefecture marks the first identification of the genus *Medaura* in China.

**New information:**

The genus *Medaura* Stål is reported for the first time from China, based on a new species *M.aculeiformis* Xie & Qian sp. n. and the identification characteristics of species are described and illustrated in this paper.

## Introduction

The stick insect genus *Medaura*, proposed by Stål in 1875, includes four valid species currently, which are distributed in Bangladesh and India. The contributions to the taxonomy of *Medaura* have been made by several researchers, including Wood-Mason 1873, Stål 1875, Wood-Mason 1875, Wood-Mason 1877, Kirby 1904, Brunner von Wattenwyl 1907, Brock and Cliquennois 2001, Delfosse 2008, Hennemann and Conle 2008, Bresseel and Constant 2017, Brock et al. 2022.

*Medaura* can be separated from closely-allied genera by the following characteristics: male head with spines absent or a very short pair of spines or tubercules between eyes; antennae shorter than fore femora. Thorax elongate, with (few to many) tubercles and/or granulations. Legs with slight dentations on femora and/or tibiae. Abdomen elongate, segments VIII-IX widened. End of anal segment incised in centre. Female head with a pair of spines between eyes; antennae less than half the length of the fore femora. Thorax smooth and robust, slightly granulated or tuberculate. Legs with minor dentation, except for mid-legs, which usually have large thorn-like lobes on the dorsal surface of thefemora and with one or two shorter thorn-like lobes on the mid-tibiae. Abdomen robust, end of anal segment incised in centre, shape variable. Operculum long, almost reaching end of anal segment. Both male and female abdomens of *Medaura* smooth to slightly granulated. Egg capsule almost oval, with capitulum and operculum with inner ring (Brock and Cliquennois 2001).

In this study, four known species of *Medaura* are reviewed, a new species collected from Mengla County, Xishuangbanna Dai Autonomous Prefecture, Yunnan Province, southwest of China is described and a list and specimen type photos of all species are provided.

## Materials and methods

Three last instar nymphs, caught in the wild, were contained in ventilated boxes with some plants inside and fed until adult. Specimens were pinned after death. All materials studied were deposited in the Insect Collection of the Southwest Forestry University, Yunnan Province, China (SWFU).

Morphological observations were made with a SOPTOP SZ stereomicroscope (Sunny Group Co., Ltd., China). Digital images were obtained using a Liyang Super Resolution System LY-WN-YH (Chengdu Liyang Precision Machinery Co., Ltd., China). Whole view images of the new specimens were taken with a Canon 5ds digital camera and LAOWA 100 mm F2.8 2X macro lens (Anhui Changgeng Optics Technology Co., Ltd., China). Stacking was done using the software Zerene Stacker (Zerene Systems LLC, USA, zerenesystems.com/cms/home). Morphological terminology follows that of Bragg (1997) and Bragg (2001).

### Acronyms for depositories

**IZCAS** - Institute of Zoology, Chinese Academy of Sciences, Beijing, China

**NHMW** - Naturhistorisches Museum Wien, Vienna, Austria

**NZSI** - National Zoological Survey of India collection, Kolcatta, India

**OUMNH** - Oxford University Museum of Natural History, Oxford, Britain

**SWFU** - Insect Collection of the Southwest Forestry University, Yunnan Province, China

## Taxon treatments

### Medaura

Stål, 1875

1BF12E49-8B11-544E-B491-034691CC97E7

Medaura : Stål 1875: 69. [as a subgenus of *Stheneboea* Stål, 1875]. -Kirby 1904: 341. [designated Stheneboea (Medaura) brunneri as type species]. -Brunner von Wattenwyl 1893: 94. [elevated to genus]. -Brock and Cliquennois 2001: 11. [revision of genus]. -Hennemann and Conle 2008: 73. [genus transferred from Clitumnini to a new tribe, Medaurini].
Menaka : Wood-Mason 1877: 342. [genus established]. -Kirby 1904: 341. [as a synonymy of *Medaura*].
Medaura Stheneboea brunneri Stål, 1875 (= *Medaurascabriuscula*)

#### List of *Medaura* species and distribution

Table 1

### Medaura aculeiformis

Xie & Qian sp. n.

E0984F1E-20BE-5BB5-ADA0-CC6C601E11C5

AF6D5C8B-DFAF-4A0C-9512-E5DD67F583D5

#### Materials

- **Type status:** Holotype. **Occurrence:** recordedBy: Jun Wang; sex: Female; lifeStage: adult; occurrenceID: 43F76939-9023-593C-BA65-DF8B02B82D08; **Taxon:** scientificName: *Medauraaculeiformis*; order: Phasmatodea; family: Phasmatidae; genus: Medaura; **Location:** country: China; stateProvince: Yunnan; municipality: Xishuangbanna Dai Autonomous Prefecture; locality: Mengla County; **Event:** year: 2021; month: 8; day: 6; **Record Level:** institutionCode: SWFU
- **Type status:** Paratype. **Occurrence:** recordedBy: Jun Wang; sex: 1 Female; lifeStage: 1 adult; occurrenceID: 764A17CD-C8D3-51BD-88CA-AA31DA7872A5; **Taxon:** scientificName: *Medauraaculeiformis*; order: Phasmatodea; family: Phasmatidae; genus: Medaura; **Location:** country: China; stateProvince: Yunnan; municipality: Xishuangbanna Dai Autonomous Prefecture; locality: Mengla County; **Event:** year: 2021; month: 8; day: 6; **Record Level:** institutionCode: SWFU
- **Type status:** Paratype. **Occurrence:** recordedBy: Jun Wang; sex: 1 Female; lifeStage: 1 nymph; occurrenceID: 78098D79-CF38-5B12-9578-6570E8588A36; **Taxon:** scientificName: *Medauraaculeiformis*; order: Phasmatodea; family: Phasmatidae; genus: Medaura; **Location:** country: China; stateProvince: Yunnan; municipality: Xishuangbanna Dai Autonomous Prefecture; locality: Mengla County; **Event:** year: 2021; month: 8; day: 6; **Record Level:** institutionCode: SWFU

#### Description

**Figure 1.**
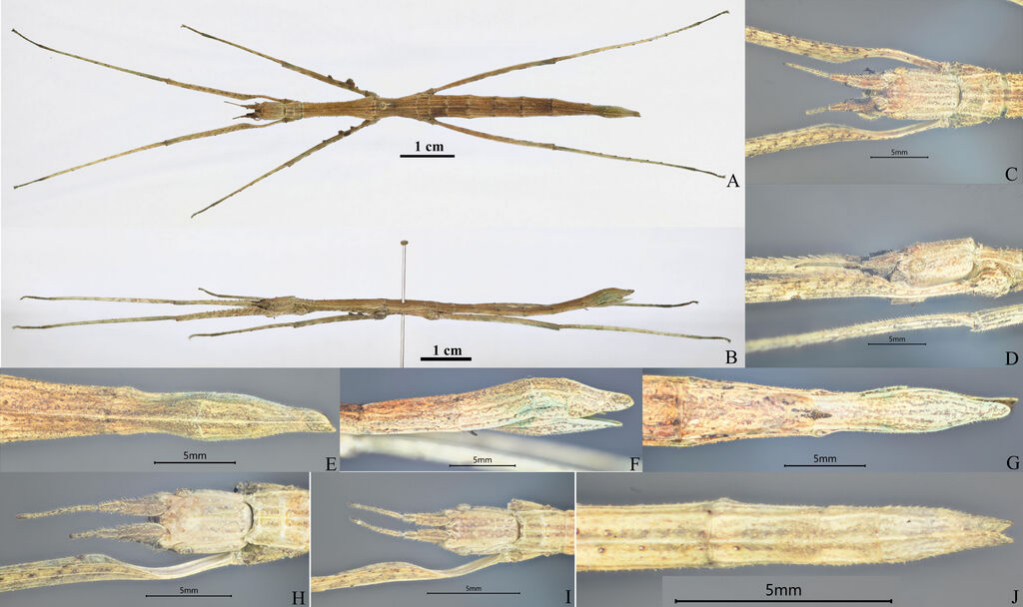
*Medauraaculeiformis* sp. n. **A-G** Holotype, female: **A** habitus, dorsal view; **B** habitus, lateral view; **C** head, dorsal view; **D** head, lateral view; **E** terminalia, dorsal view; **F** terminalia, lateral view; **G** terminalia, ventral view; **H-J** Paratypes, females ♀; **H** head, dorsal view, adult; **I** head, dorsal view, nymph; **J** terminalia, dorsal view, nymph.

**Female.** Medium-sized. Body slender. General colouration brown (Fig. 1 A-B).

**Head.** Squarish, longer than wide, vertex slightly concave and with two small granules between compound eyes. Compound eyes rounded, occupying 1/4 of the genae. Occiput centre slightly convex, covered with sparse and small granules (Fig. 1C, D, H and I). Antennae filiform, 14 segments, shorter than half the length of fore femora; scapus rectangular and flattened, longer than pedicellus, almost 3x length of pedicellus; pedicellus flattened oval and longer than the third segment (Fig. 1H and I). **Thorax.** Pronotum nearly rectangular, slightly longer than width, almost 1/2 length of head, with sparse small granules; transverse and longitudinal sulci crossing at middle area and distinctly. Mesonotum longer than width, anterior narrowed and gradually widened posteriorly, about 4.5x length of pronotum; with distinct median longitudinal carina and sparse small granules; a row of small granules on the lateral carina of mesonotum. Metanotum nearly rectangular, almost 2/3 length of mesonotum, median area narrow, median longitudinal carina distinct; 2-3 small granules on the lateral carina of metanotum (Fig. 1A and B). **Abdomen.** Cylindrical, with slightly granulated, median longitudinal carina distinct. Median segment rectangular, wider than length, almost 1/4 length of metanotum, obviously segmented. Tergum II-VI robust and each segment gradually becoming longer. Tergite VII gradually narrowed. Tergite VIII narrowed in median area and broadened posteriorly, about 3/4 length of tergite VII. Tergite IX about 1/2 length of tergite VIII. Tergites II-IX with distinct lateral carina on both sides of median longitudinal carina. Anal segment gradually narrowing, about 1.5x length of tergite IX, end of anal segment with a deeply V-shaped incision in centre (Fig. 1A, B, E-G and J). Sternite VII with a distinct needle-like praeopercular organ (Fig. 1B, F and G). Operculum not surpassing posterior margin of anal segment, scoop-shaped and tapering posteriorly (Fig. 1E-G). Cerci short, slightly lanceolate and hidden beneath anal segment (Fig. 1H). **Legs.** All long and moderately slender, covered with sparse and short bristles; profemora distinctly curved basally, with minor serrations in dorsal carina; mesofemora with five round-lamellar lobes; metafemora smooth. Pro- and meta-femora shorter than corresponding tibiae; mesofemora about as long as mesotibiae. Protibiae smooth; meso- and meta-tibiae with a few small serrations (Fig. 1A and B).

##### Measurements(mm)

Table 2

#### Diagnosis

**Figure 3.**
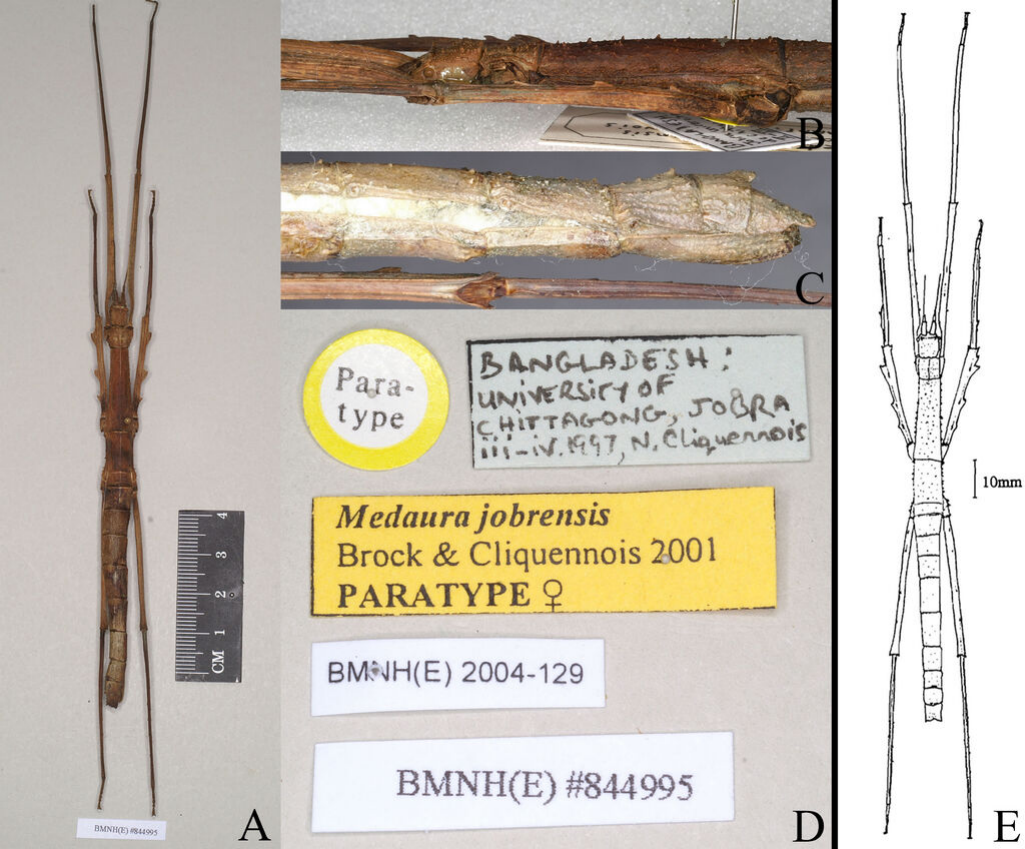
*Medaurajobrensis* Brock & Cliquennois, 2001. **A-D** Paratype, female (from Phasmida Species File 2022, photos by Paul Brock, published under CC BC -ShareAlike 4.0 International Licence): **A** habitus, dorsal view; **B** head and thorax, lateral view; **C** end of abdomen lateral view; **D** female, paratype data labels; **E** habitus, dorsal view (Modified from Brock and Cliquennois 2001).

**Figure 4.**
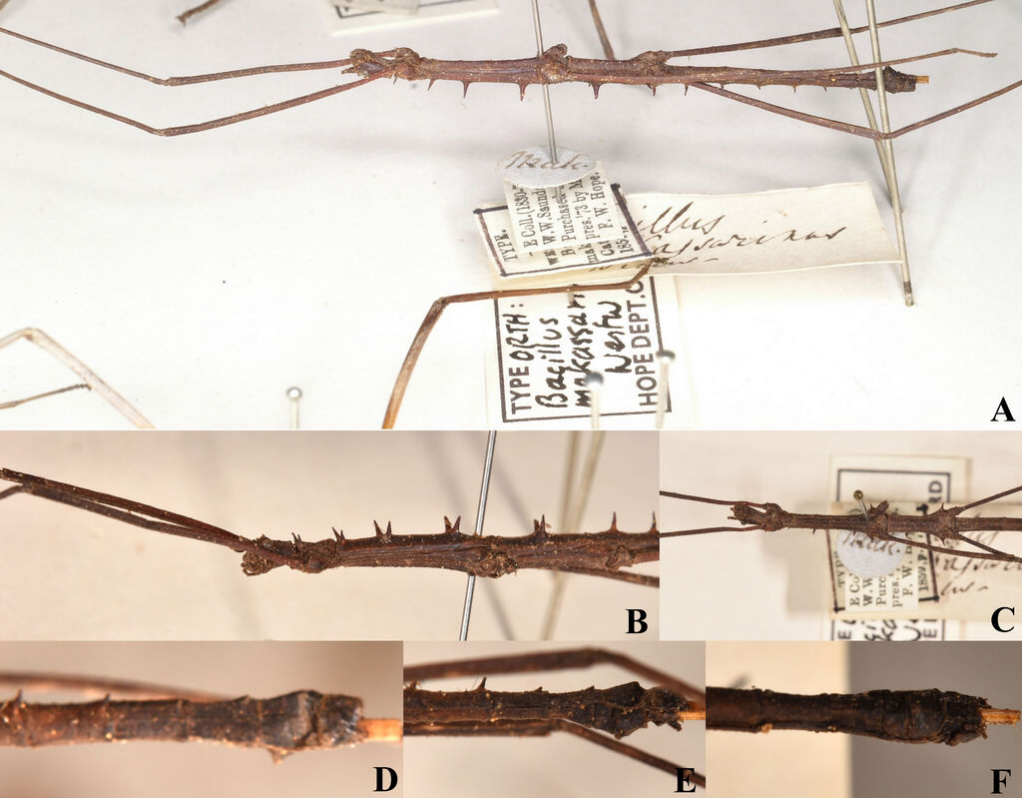
*Medauramakassarinus* (Westwood, 1859). **A-F** Holotype, male (from Phasmida Species File 2022, photos by Paul Brock, published under CC BC - ShareAlike 4.0 International Licence): **A** habitus, lateral view; **B** head and thorax, lateral view; **C** head and thorax, ventral view; **D** end of abdomen, dorsal view; **E** end of abdomen lateral view; **F** end of abdomen, ventral view.

The new species is similar to *M.scabriuscula* and *M.jobrensis*, but can be separated by the fewer antennal segments, ornamentation between the compound eyes, serrations or lobes on legs and the incised shape at end of the anal segment. The new species with two small granules between the compound eyes; antennae 13-14 segments; mesofemora with five round-lamellar lobes and mesotibiae with few tiny serrations; anal segment longer than 9^th^ segment and end with deep and narrowed V-shaped incision in centre (Fig. 1). In *M.scabriuscula*, two bold spines between the compound eyes; antennae 18-19 segments; mesofemora with three large dentate foliaceous lobes dorsally and three small spines on the central carina, mesotibiae with two smaller foliaceous lobes at the proximal end; anal segment longer than 9^th^ segment and tip boldly triangular incised in centre, giving it the appearance of having two leaf-like lobes. In *M.jobrensis*, with two bold spines on raised ridge between the compound eyes; antennae 21-22 segments; mesofemora with three large dentate foliaceous lobes dorsally and three small spines on the central carina, mesotibiae with two smaller foliaceous lobes at the proximal end and other minor spines; anal segment same length as 9^th^ segment and tip slightly and unevenly triangularly incised in centre; end of 9^th^ segment with large twin tubercles in centre (Figs 3, 4; Brock and Cliquennois 2001).

#### Etymology

The name (lat. *aculeiformis* = needle-like) refers to the needle-like praeopercular organ on sternite VII of female.

#### Distribution

China (Yunnan).

#### Notes

Antennal segments of new species have 14 in adult, 13 in the last instar nymph. Three last instar nymphs fed on *Rosachinensis* Jacq. (Rosaceae) and *Nephrolepisauriculata* (L.) Trimen (Nephrolepidaceae) in the lab, but these may not be the real host plants. One nymph died, two nymphs turned into adults and died soon afterwards without spawning. Adults bite each other, causing the posterior margin of the anal segment to be incomplete. Thus, the egg is unkown.

#### Type photos

Fig. 1

### Medaura austeni

(Wood-Mason, 1875)

CF6F2D53-D226-5CF2-BE9C-F24B37B602D4

*Lonchodesausteni*: Wood-Mason 1875: 216. [original description].
*Promachusausteni*: Kirby 1904: 326. [transferred genus].
*Medauraausteni*: Brunner von Wattenwyl 1907: 241. [transferred genus]. Mandal and Yadav 2010: 31. [Redescribed from literature].

#### Diagnosis

As the type specimen of *Medauraausteni*, deposited in NZSI, has been lost (Mukherjee and Sirinivasan 2013), we did not provide the photos here. From literature, only the male characters were described including: head and thorax with minute granules; mesonotum, metanotum and abdomen with carina and strong spines; anal segment small, fused at base, rounded; subgenital plate compressed and hood-shaped; middle and posterior femora at base below with single spine (MandaI and Yadav 2010).

### Medaura jobrensis

Brock & Cliquennois, 2001

7835BC37-8C90-529A-A88E-7A498DB784CB

*Medaurajobrensis*: Brock and Cliquennois 2001: 19. [original description] ­Delfosse 2008: 3 [biology]. ­Brock et al. 2016: 178. [type data].

#### Diagnosis

The type specimen of *Medaurajobrensis* was deposited in NHMW. The original literature provides a detailed description of male, female and egg (Brock and Cliquennois 2001).

#### Type photos

Fig. 2, Fig. 3

### Medaura makassarinus

(Westwood, 1859)

36EF8108-0531-572A-85E3-33925C68BC03

*Bacillusmakassarinus*: Westwood 1859: 179. [original description].
*Medauramakassarinus*: Kirby 1904: 341. [transferred genus].

#### Diagnosis

The type specimen of *Medauramakassarinus* was deposited in OUMNH. As only the male was simply described in literature, we organised photos of the male from PSF, but unfortunately the photo of the abdomen dorsal view is unclear.

#### Type photos

Fig. 4

### Medaura scabriuscula

(Wood-Mason, 1873)

FCEAB040-6362-52D6-8989-A04505102B32

*Bacillusscabriusculus*: Wood-Mason 1873: 55. [original description].
*Menakascabriuscula*: Wood-Mason 1877: 342. [transferred to new genus].
*Medaurascabriusculus*: Kirby 1904: 341. [catalogue of species]. ­Brock and Cliquennois, 2001: 15. [redescription, first description male and egg, synonymy]. Delfosse 2008: 3. [biology].
Synonym: Stheneboea (Medaura) brunneri: Stål 1875: 69. [original description]. Wood-Mason 1877: 342. [listed as a synonym of *Menakascabriuscula*]. Kirby 1904: 341. [type species designation, listed as synonym of *Medaurascabriusculus*].
Synonym: *Medauranimia*: Brunner von Wattenwyl 1907: 241. [original description]. Brock 1998: 46. [type data]. Brock and Cliquennois 2001: 15. [listed as synonym of *Medaurascabriuscula*].
Synonym: *Medaurasubintegra*: Carl 1913: 1. [original description]. Brock and Cliquennois 2001: 15. [listed as synonym of *Medaurascabriuscula*].

#### Diagnosis

All type specimens of *Medaurascabriuscula* were deposited in NZSI, NMW and MHNG. However, only the female was simply described in the original literature. Subsequently, Brock and Cliquennois (2001) described the male and provided a supplementary description of the female and egg.

#### Type photos

Fig. 5

## Discussion

The stick insect genus *Medaura* is recorded for the first time in China and now 10 genera of Medaurini are distributed in the country. The new species was collected in Mengla County of Xishuangbanna Dai Autonomous Prefecture in the south of Yunnan, its borders with the northwest of Laos and neighbouring Burma in the west belonging geographically to the transition zone of Asian mainland and the southeast Asia peninsula. The climate in Mengla is humid tropical monsoon. The other four known species of *Medaura* are also principally tropical in distribution. The distribution of *Medaura* is now further to the north since *M.aculeiformis* sp. nov. has been found.

China is one of the diversity hotspots in the world because of the highly complex topography. The biodiversity and endemism of phasmids occupy high proportions in the south-western mountainous regions, the taxa showing higher diversity in southern China, tropical regions bordering Vietnam, Thailand or Myanmar in particular (Hennemann et al. 2008). Yunnan belongs to the Oriental Region and recorded 30 genus and 137 species of stick insects at present (through the generalisation of the literature data). Xishuangbanna as the south China mountain subregion is one of the most abundant species regions in China; it has important implications for investigating stick insects in this area more thoroughly.

There are still some problems in *Medaura* that have not been well solved. Regarding the taxonomic position of *M.austeni* and *M.makassarinus*, we agree with Brock and Cliquennois's view (2001) that these two species may not belong to *Medaura*. However, we think that more collecting is needed to obtain more adult and egg specimens in order that we can better discuss the taxonomic position. This problem also applies to other genera of Medaurini; if sufficient material is available, molecular methods can be applied to help solve some problems of Chinese Phasmatodean in future studies.

## Supplementary Material

XML Treatment for Medaura

XML Treatment for Medaura aculeiformis

XML Treatment for Medaura austeni

XML Treatment for Medaura jobrensis

XML Treatment for Medaura makassarinus

XML Treatment for Medaura scabriuscula

## Figures and Tables

**Figure 2.**
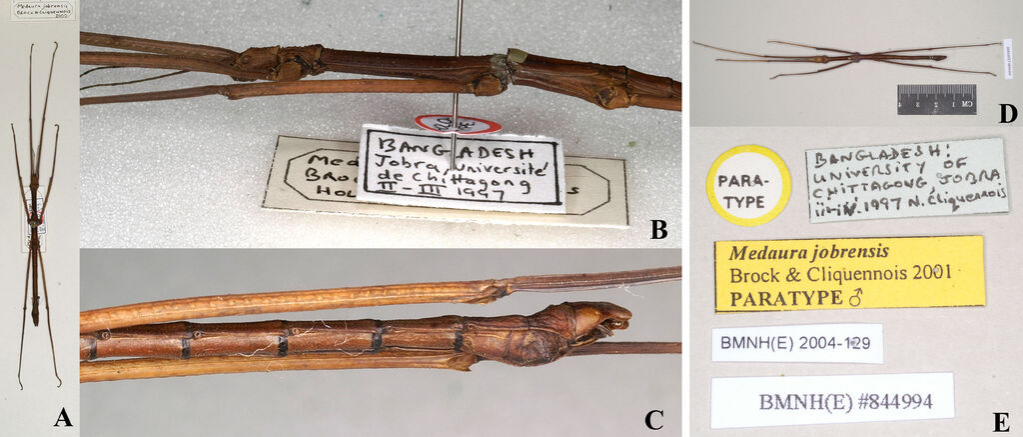
*Medaurajobrensis* Brock & Cliquennois, 2001. **A-C** Holotype, male (from Phasmida Species File 2022, photos by Paul Brock, published under CC BC -ShareAlike 4.0 International Licence): **A** habitus, dorsal view; **B** head and thorax, lateral view; **C** end of abdomen, lateral view; **D & E** Paratype, male (from Phasmida Species File 2022, photos by Paul Brock, published under CC BC -ShareAlike 4.0 International Licence): **D** habitus, dorsal view; **E** paratype data labels.

**Figure 5.**
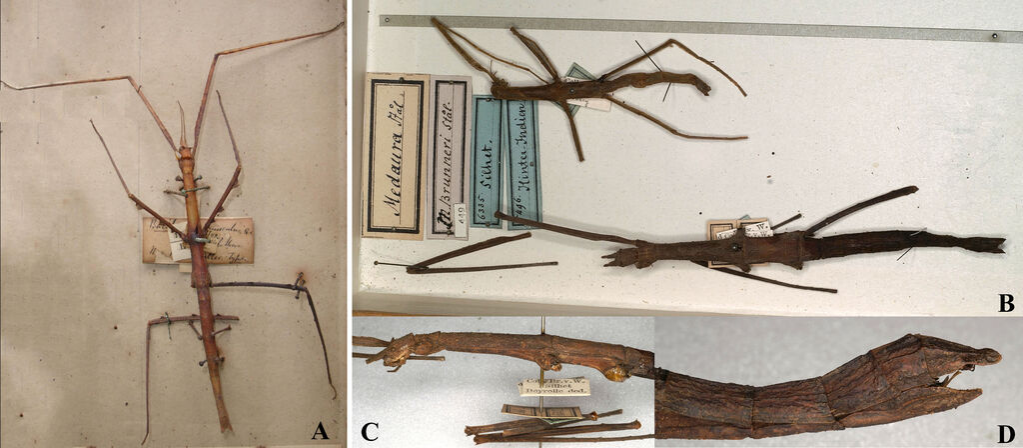
*Medaurascabriuscula* (Wood-Mason, 1873). **A** female, holotype; **B-D** female, show the holotype of the synonym Stheneboea (Medaura) brunneri (all from Phasmida Species File 2022. **A** photo by Tushar Mukherjee, **B-D** photos by Paul Brock, published under CC BC -ShareAlike 4.0 International Licence): **A** habitus, dorsal view; **B** habitus, dorsal view; **C** head and thorax, lateral view; **D** end of abdomen lateral view.

**Table 1.** List of the genus *Medaura* of species and distribution.

Species	Female	Male	Egg	Distribution	Notes
*M.aculeiformis* Xie & Qian sp. n.	known	unknown	unknown	China	
*M.austeni* (Wood-Mason, 1875)	unknown	known	unknown	India	Loss of type specimen
*M.jobrensis* Brock & Cliquennois, 2001	known	known	known	Bangladesh	
*M.makassarinus* (Westwood, 1859)	unknown	known	unknown	Indonesia (Sulawesi)	
*M.scabriuscula* (Wood-Mason, 1873)	known	known	known	India	Type-species

**Table 2.** Measurements of *Medauraaculeiformis* Xie & Qian sp. n. (mm).

Length (mm)	Holotype	Paratype	
	♀	♀	♀(nymph)
Body	69.5	57.2	41.8
Head	6.3	4.7	3.3
Antennae	6.5 (incomplete)	6.9	5.7
Pronotum	3.3	3.0	2.1
Mesonotum	13.8	12.0	8.4
Metanotum	7.9	7.2	5.5
Median segment	2.9	2.5	1.7
Profemora	22.7	18.3	13.8
Mesofemora	15.7	13.1	9.3
Metafemora	21.4	17.1	10.0
Protibiae	24.1	missing	15.0
Mesotibiae	16.0	12.9	9.1
Metatibiae	23.7	19.4	10.4
